# Accuracy and Misleadingness of Anatomical and Embryological Statements in State‐Level Abortion Ban Legislation in the United States

**DOI:** 10.1111/psrh.70001

**Published:** 2025-02-28

**Authors:** Rachel N. Feltman, Steven R. Lewis, Nathan E. Thompson

**Affiliations:** ^1^ Department of Anatomy NYIT College of Osteopathic Medicine Old Westbury New York USA; ^2^ Department of Clinical Medicine NYIT College of Osteopathic Medicine Jonesboro Arkansas USA

**Keywords:** abortion, domestic policy, law/legal issues, public health, United States

## Abstract

**Objective**: In the last 15 years, the United States has seen a surge in anti‐abortion legislation enacted at the state level. Many of these pieces of legislation utilize anatomical and embryological details to justify the necessity of abortion bans. In this study, we evaluated the level to which these statements are accurate and/or misleading, if at all, as determined by experts in anatomy and embryology.

**Methods**: Experts evaluated statements of anatomical and embryological fact included in Legislative Findings (or equivalent) sections of state‐level abortion ban legislation passed between January 2016 and January 2023 on their level of accuracy and misleadingness. We investigated 56 pieces of legislation from 23 states, which resulted in 57 testable statements common to 13 pieces of legislation across 12 states. Forty‐one experts in anatomy and embryology rated each statement from 1 (completely inaccurate/misleading) to 5 (completely accurate/non‐misleading).

**Results**: Mean accuracy for all 57 statements was 3.0 ± 1.2 (range: 1.4–4.3) and the overall level of misleadingness was 2.5 ± 1.2 (range: 1.3–3.8).

**Conclusion**: All 57 statements were significantly different from a null expectation of completely accurate and completely non‐misleading. Statements made about anatomy and embryology aim to justify abortion bans but contain, to varying extents, inaccurate and misleading information, thereby contributing to the detrimental effects of restrictive abortion legislation on the health and well‐being of pregnancy‐capable people.

## 1 Introduction

Scientific justifications for restricting abortion rights have increased in the last several decades. While the United States (US) Supreme Court decision in *Roe v. Wade* (*Roe*) enshrined the right to an abortion up to the point of fetal viability [1], the 1992 Supreme Court decision in *Planned Parenthood v. Casey (Casey)* allowed states to impose further restrictions on the right to abortion as long as they did not create an undue burden [2]. Unfettered state‐level autonomy to restrict abortion access—up to and including banning all abortions—was allowed with the 2022 overturning of *Roe v. Wade* in *Dobbs v. Jackson Women's Health Organization* (*Dobbs*) [3].

Following *Casey*, U.S. state‐level legislative efforts to restrict abortion access focused on several areas, including facilities regulations, parental involvement, mandatory counseling and waiting periods, and most rigidly, timing‐based bans on abortion care [4]. The patchwork of state‐level restrictions on access to, and denial of, abortion care following *Roe*, *Casey*, and *Dobbs* has led to an increase in maternal, infant, and fetal mortality, mental health inequities among women, and an increase in life‐threatening consequences for women denied abortion care [5, 6, 7, 8, 9, 10, 11, 12, 13, 14, 15]. These laws also undermined the ability of patients to make informed decisions [16, 17]. This is in part due to the propagation of inaccurate or misleading medical information that results from them [16]. For instance, in 2016, Daniels et al. [16] found that nearly one‐third of all “factual” information in state‐mandated and authored informational material provided to patients was inaccurate, with that level being much higher for information regarding the early weeks of pregnancy.

In this study, we investigated the factual underpinnings of justifications for recent US state‐level legislation. We utilized a similar approach to Daniels et al. [16] and focused specifically on legislation banning abortion care. Abortion bans more restrictive than those allowed under *Roe* and *Casey* have rapidly proliferated following the *Dobbs* decision [14]. Many states include within their legislation a section of “Legislative Findings,” “Fact,” “Purpose,” or “Intent.” Encompassed therein are the purported factual statements that are the justification for legislative action. For legislation banning abortion care, many of these statements are of anatomical and embryological “fact.” However, embryology, anatomy, and development are complex topics, the level of accuracy of this information is unknown, and “factual” statements within legislation may allow misinformation to be used to justify restrictions on abortion care. Scientific accuracy in public policy is crucial to ensure that a reliable foundation guides decision‐making, prevents harm and builds trust in the healthcare system, and leads to the best outcomes for individuals and society.

## 2 Methods

### 2.1 Approach

**FIGURE 1:**
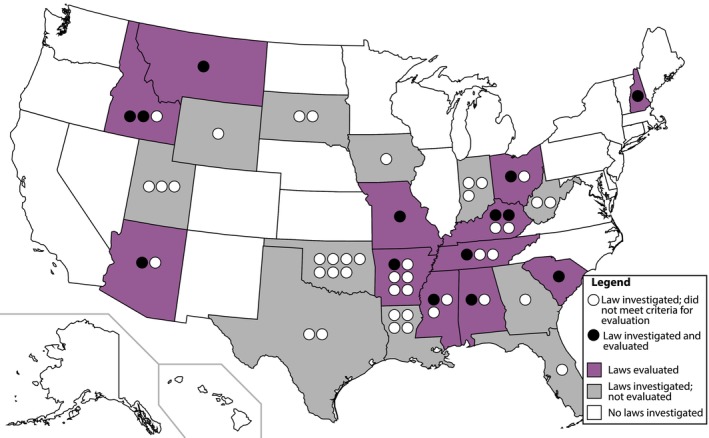
Pieces of legislation investigated by state, indicating those that were investigated, but did not meet the criteria for inclusion (gray shading, white circles) and those that were investigated and included in the analysis (purple shading, black circles).

We compiled all state‐level legislation passed and enacted between January 2016 and January 2023 using the Guttmacher Institute Legislation Tracker [4], specifically under the category of “Abortion Ban.” In total, 56 pieces of legislation from 23 states were investigated (Figure 1 and Table S1). We retained in our analysis those pieces of legislation that included anatomical or embryological statements of fact within a “Legislative Findings” or similar section (Table S2). Within each “Legislative Findings” (or similar) section, we isolated each statement of anatomical or embryological fact as a survey item. We evaluated each statement using a 4‐prong evaluation for testability (see Data S1: Supporting Methods), coalesced statements across states (Tables S1–S3), and edited statements into a standard format which retained the intent of the statement (see Data S1: Section 1 for details; Table S4). This resulted in 13 pieces of legislation from 12 states and 57 unique statements (Figure 1). Broadly, the statements covered general development (25), neurological development (26), and some miscellaneous topics (6).

Participants rated each statement's accuracy (1 = completely inaccurate; 5 = completely accurate) and level of misleadingness (1 = completely misleading; 5 = completely non‐misleading) on a five‐point scale [16]. Participants also rated their level of familiarity with, and confidence in, early embryology and human anatomy on a 0–10 point scale. Finally, participants reported their current professional affiliation(s) and demographic characteristics including gender, political party, ideology, religious affiliation, religious level, self‐described racial identity, and highest educational level. Ethical approval was obtained by the NYIT Institutional Review Board, which determined this project to be exempt (BHS‐1828).

### 2.2 Evaluators of Accuracy and Misleadingness

We recruited experts in human anatomy and embryology to complete the survey and distributed it to participants in three ways: (1) posted on the American Association for Anatomy (AAA) online forum (*n* = 15 opened surveys), (2) sent to AAA members who had listed their email in the association directory (*n* = 565 emails), and (3) emailed to professors of anatomy departments across the country (*n* = 110 emails). The research team (NET and RNF) verified all participants who completed the survey as subject matter experts. We defined this as a participant who has either taught or conducted primary research in anatomy or embryology at an institute of higher education and determined this via public record searches (e.g., institutional or personal research webpages).

### 2.3 Statistical Analysis

We tested each statement for significant difference from a null expectation of 5 (completely accurate or completely non‐misleading) using two‐tailed Wilcoxon Rank Sum one‐sample tests. Where possible, we tested for associations between participant demographic characteristics as well as familiarity and confidence in anatomy and embryology with mean statement response using non‐parametric Spearman rank correlations and/or Kruskal–Wallis rank sum tests (see Data S1: Section 2 for additional details). Finally, in order to evaluate self‐selection bias, we also tested the distribution of participant demographic characteristics (gender, political party, ideology, and religious affiliation) against estimated demographics for US academics [18, 19] using Fisher's exact tests (see Data S1: Section 2 for details).

## 3 Results

In total, 41 participants fully completed the survey out of 96 that began it (42.8% completion rate following CHERRIES guidelines [20]). Participants self‐reported their familiarity and confidence with early embryology as 7.8 ± 2.3 and 7.9 ± 2.1 (respectively) and their familiarity and confidence in human anatomy as 9.6 ± 0.8 and 9.6 ± 0.8 (respectively). Participant demographics are provided in Table 1. The demographic characteristics of participants herein were not significantly different from what would have been expected based on nationwide demographic characteristics of US‐based college and university professors (Table 1).

**TABLE 1:** Respondent demographic characteristics and associations with scoring of accuracy and misleadingness of statements.

			Sample bias	Association with mean accuracy		Association with mean misleadingness	
	Study participant characteristics (*n* = 41)	Estimated study characteristics (*n* = 41)	*p*	*p*	*ρ*	*p*	*ρ*
Gender			0.10	0.95	0.01	**0.01**	−0.39
Female	18	11					
Male	21	30					
Prefer not to say	1	—					
Political party			0.36	**0.02**	0.38	**0.01**	0.41
Strong democrat	15	13					
Weak democrat	5	8					
Independent‐lean democrat	7	8					
Independent	8	3					
Independent‐lean republican	2	3					
Weak republican	1	4					
Strong republican	0	2					
Don't know/refused	2	—					
Ideology			0.33	**0.009**	0.43	0.14	0.25
Liberal	23	18					
Moderate	12	19					
Conservative	3	4					
Don't know/refused	2	—					
Religious affiliation			0.09	0.16^#^	—	0.14^#^	—
Not affiliated or nothing in particular (atheist, agnostic)	28	21					
Catholic	4	4					
Protestant	2	7					
Other Christian	3	0					
Jewish	2	6					
Other non‐Christian	1	2					
Religious level			—	0.07	0.30	0.38	0.15
Not religious	24	—					
Slightly religious	6	—					
Moderately religious	7	—					
Very religious	2	—					
Don't know	2	—					
Self‐described racial identity			—	—	—	—	—
White	36	—					
Black or African American	1	—					
Prefer not to say	3	—					
Highest educational level			—	—	—	—	—
Doctorate degree	40	—					
Master's degree	1	—					
Embryology familiarity, mean (SD)	7.8 (2.3)	—		0.94	0.01	0.89	−0.02
Embryology confidence, mean (SD)	7.9 (2.1)	—		0.86	0.03	0.89	0.02
Anatomy familiarity, mean (SD)	9.6 (0.8)	—		0.42	0.13	0.44	0.13
Anatomy confidence, mean (SD)	9.6 (0.8)	—		0.74	0.05	0.41	0.14

*Note:* Sample bias indicates whether a significant difference exists between estimated academic demographic characteristics and those among our study participants (see Section 2 in Data S1). *p* Values for associations between responses and mean accuracy and misleadingness are from Spearman rank correlations, except for religion (#) which are from Kruskal–Wallis rank sum tests. Bold values indicate significance.

**FIGURE 2:**
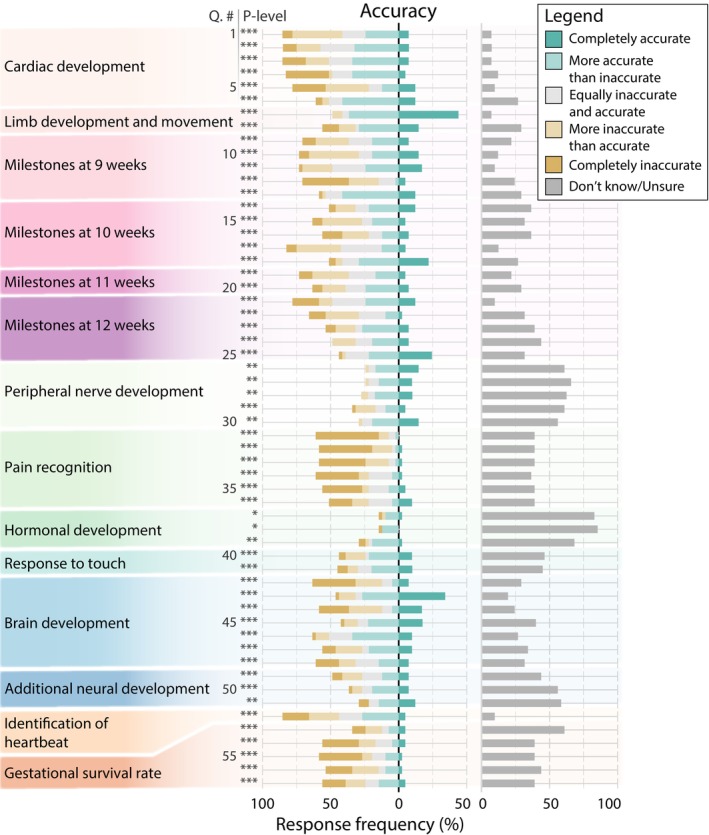
Accuracy of all 57 statements grouped into thematic category. Data is presented as frequency of participant responses. Proportion of responses of “completely accurate” are presented to the right of the black line while all others are on the left. Responses of “don't know/unsure” are on the right in gray. A higher proportion of green responses, especially to the right of the solid black line, indicate a question was scored as more accurate. The total response frequency for each question sums to 100%. Asterisks represent significant differences from a score of 5 (completely accurate) at the *p* < 0.05 (*), *p* < 0.01 (**), or *p* < 0.001 (***) level. Individual statement text and statistics are provided in Table S1.

**FIGURE 3:**
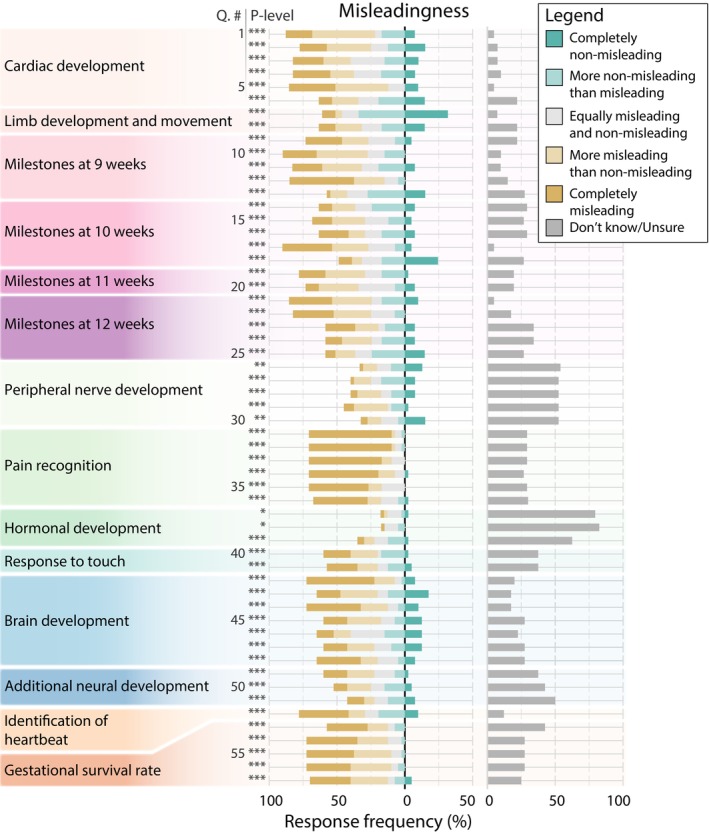
Misleadingness of all 57 statements grouped into thematic category. Data presentations and symbols as in Figure 2.

Mean accuracy for all statements was 3.0 ± 1.2 (range: 1.4–4.3; Figure 2) and the overall level of misleadingness was 2.5 ± 1.2 (range: 1.3–3.8; Figure 3). Statements regarding early fetal development were most accurate and non‐misleading (Q1–25; accuracy 3.1 ± 1.2 and 2.7 ± 1.3, respectively), followed by statements of neurological development (Q26–51; accuracy: 3.1 ± 1.2; misleadingness: 2.4 ± 1.2), then by six miscellaneous statements (Q52–57; accuracy: 2.4 ± 1.3; misleadingness: 1.9 ± 1.1). All statements evaluated were significantly less than 5.0 for both accuracy and misleadingness (*p* < 0.05; Figures 2 and 3; Table S4).

Statement scoring was correlated with some demographic characteristics. Gender was correlated with misleadingness, with women scoring statements as more misleading (*p* < 0.05). While only three participants identified as Republican or with conservative ideology, their results suggest a correlation between political party and/or ideology and response. Republicans rated statements as more accurate and non‐misleading and conservatives rated statements as more accurate.

## 4 Discussion

We found that all statements of anatomical and embryological fact contained within restrictive state‐level abortion legislation were, to varying extents, inaccurate and misleading. For all statements, accuracy and misleadingness were significantly different from our null expectation of completely accurate/non‐misleading. The subset of statements that were found to be the most accurate were those of limb development and movement and peripheral nerve development. Statements of limb development and movement were also found to be the most non‐misleading. Statements regarding pain recognition were both the most inaccurate and misleading.

The results found here parallel several previous investigations on the use of medical language in the abortion legislation landscape. Prior work has found misrepresentation of medical information used as a strategy of anti‐abortion advocates during expert testimony periods of state‐level legislative debates [21, 22], and as an outcome of state‐level laws that mandate information be given to patients [16]. Indeed, Daniels et al. [16] found that less than half of the anatomical “facts” in state‐mandated informed‐consent material were completely accurate (42%) or completely non‐misleading (47%). Taken together, restrictive abortion legislation therefore not only leads to faulty information being presented to patients [16], but faulty information may be used to justify the passing of legislation itself (herein and [21, 22]).

A perceived limitation of this study might be the small number of experts who scored statements (41 participants). This was somewhat expected as embryological development is a complex field and the survey mechanism used herein was necessarily long. The only similar study evaluating state‐mandated informed consent abortion material utilized a panel of seven experts, drawn from the same population as herein (American Association for Anatomy members) [16]. Furthermore, our study shows that some demographic characteristics (gender, political party, and ideology) may influence how experts rate accuracy and misleadingness (Table 1). This is not particularly surprising given that gender, political party, and ideology are known to be correlated with views on abortion, with men, Republicans, and conservatives having more negative attitudes towards abortion [23, 24, 25, 26, 27]. It is notable that the effects of demographic characteristics were not uniform: gender had an effect on misleadingness, ideology had an effect on accuracy, and political party had an effect on both. The latter two should be interpreted with some caution given that only three participants were of conservative ideology or Republican to any degree. However, these results do suggest that demographic characteristics may play a role in how legislative wordings are crafted, in particular given that state‐level legislatures themselves are dominated by men [28].

In this study, we analyzed all pieces of state‐level legislation that contained purportedly factual anatomical/embryological statements used to justify restrictive abortion legislation. We did not discriminate pieces of legislation based on their restrictive outcome; that is, whether the outcome would have been allowed under *Roe*, *Casey*, or *Dobbs*. It is noteworthy that even the least restrictive, viability‐based bans allowed under *Roe* have been subject to debate regarding the accuracy/inaccuracy of the concept of viability [29, 30, 31] and even whether an accurate, discrete timepoint of viability can be defined at all [30, 32]. Our study did include a few statements that had been used in support of mid‐gestation age abortion bans, specifically following a trend of using nervous system development and pain perception as a marker of viability [30]. Interestingly, our data show that neurological statements, and specifically pain recognition statements (Figures 2 and 3), tended to score low for both accuracy and non‐misleadingness. That being said, the vast majority of statements evaluated herein concerned timepoints that were well before any possible definition of viability.

The results of this study have many implications for the health and well‐being of women seeking abortion care. Legislation restricting abortion care and access has many detrimental effects, including increased maternal, fetal, and infant mortality rates [5, 6, 7, 8, 13, 15]. Women unable to access abortion care experience more life‐threatening conditions and long‐term negative health consequences [10, 12, 14, 33] as well as socioeconomic consequences, including lower levels of financial insecurity and economic opportunity [34], among other harms [11]. Furthermore, restrictive abortion legislation exacerbates existing social and medical inequities [35, 36] and disproportionately affects the most vulnerable populations of women [37].

Given the health and socioeconomic harms that result from restrictive abortion bans, it is imperative that the factual basis of legislation be as completely accurate and non‐misleading as possible. Current state‐level legislation banning abortion does not appear to meet that threshold.

## Conflicts of Interest

The authors declare no conflicts of interest.

## Supporting information


**S1.** Supporting Information.

**Data S2.** Supporting Tables.

## Data Availability

All data is included as Supporting Information Tables S1–S5.
