# Supplementary material for: Accuracy and Misleadingness of Anatomical and Embryological Statements in State‐Level Abortion Ban Legislation in the United States
Source: Perspect Sex Reprod Health. 2025 Feb 28;57(1):17–24. doi: 10.1111/psrh.70001 (PMC11936854; doi:10.1111/psrh.70001)
Supplement: Supplementary file 1 — S1. Supporting Information. [file PSRH-57-17-s001.docx]

**Supporting Information for:**

Accuracy and misleadingness of anatomical and embryological statements in state-level abortion ban legislation

Rachel N Feltman, Steven R Lewis, Nathan E Thompson

Address correspondence to NET (nthomp03@nyit.edu)

**This PDF file includes:**

Supporting Methods

Legends for Supporting Tables S1 to S5

SI References

**Other supporting materials for this manuscript include the following:**

Supporting Tables S1 to S5 (.xlsx file)

Supporting Methods

**S1: Survey Creation.** The initial list of state legislation investigated included 56 pieces of legislation from 23 states (Supporting Table S1). Of these initial 56 pieces of legislation, many did not contain any anatomical or embryological facts within the legislative findings, facts, or purposes (or equivalent) section. One piece of legislation had anatomical/embryological statements in the introduced and engrossed version (MS SB2116; 2019), but not in the final adopted version and we thus excluded it. Another piece of legislation (ID SB1309, 2022) was an amendment to prior legislation (ID HB366, 2021), and thus both had the exact same statements. We counted this as one piece of legislation. Finally, we included only statements within the legislative findings, facts, or purposes (or equivalent) section. Notably, several pieces of legislation have a section requiring provision of ‘informed consent’ material; we excluded any facts within those mandate-specific sections (e.g. IN HB1217; IN HB1337; GA HB481). We did this for three reasons 1) these facts are not those being used as a predicate for legislative action, 2) the level of accuracy and misleadingness in informed consent material has already been documented,^1^ and 3) when present, those statements often mirrored the language and statements used elsewhere and thus many of those statements would nevertheless be tested.

For a statement to then be included into the final survey mechanism, each statement had to: 1) be a statement of anatomical or embryological fact, 2) be falsifiable, 3) refer to a specific timepoint(s) in development, and 4) not require specialized knowledge outside of normal developmental expertise (e.g. in-utero surgical experience). An initial application of these criteria resulted in 13 pieces of legislation from 12 states. The statements of anatomical or embryological fact presented in these pieces of legislation are in Supporting Table S2. We discretized and compiled these statements into initial survey statements (n=155). Many of these statements are, or are nearly, duplicates across states. We then aligned all statements by category and type (Supporting Table S3) and identified identical statements, or statements that referred to the same underlying fact. For identical statements, nearly identical statements, or statements that referred to the same underlying fact we retained only one exemplar statement (bolded statements in Supporting Table S3). In some cases, states utilized slightly different wording that changed the underlying fact. For instance, the statement ‘At nine weeks gestation, teeth are present’ is common to AZ SB1164, MS HB 1510, and KY HB3. However, AR HB 1439 utilizes the statement ‘At nine weeks gestation, buds for teeth are present’. If any verbiage caused a statement to have a potentially different meaning, we included it as a separate statement. At this stage, we excluded another 19 of the initial 155 statements based on the four qualifying criteria previously listed.

Accounting for similar statements resulted in the final list of 57 statements, which we organized into statements of general development, neurological development, and another small set of miscellaneous statements. We further standardized the wording and grammar of the statements across the survey. In most cases, this involved only minor grammatical changes. However, for many statements there was variation as to how the embryo and/or fetus was referred, if necessary. For all statements where it was necessary to refer to the embryo/fetus, we adopted the phrasing “An unborn human being…”. We made this decision as this verbiage was used by most of the states investigated (Supporting Table S3), and that other, alternative verbiage appeared to be more specific and/or emotionally charged (e.g. “An unborn child…”). For many of the final statements, the statement is a complete sentence. However, for two groups of neurological statements, the differences between statements across pieces of legislation referred to differences in the timing of development. For these two groups of statements, the survey presented a stem clause (“Peripheral cutaneous sensory receptors…”), followed by the relevant end clauses, and the participants evaluated each on the 5-point scale for accuracy and misleadingness. All final statements, the state which utilizes that fact, and final survey number and statistics are presented in Supporting Table S4. Full deidentified results provided in Redcap labelled format are presented in Supporting Table S5.

**S2: Demographic data and statistical analysis.** We analyzed associations between survey responses and demographic characteristics by first calculating each expert’s mean response for all questions (excluding ‘don’t know/unsure’ responses) for both accuracy and misleadingness. For those demographic factors that were presented as logically ordered/continuous variables (e.g. political party, ideology, and religious level), we performed correlations between mean response and demographic characteristic using non-parametric Spearman rank correlations (Table 1). We also tested gender using Spearman rank correlations as all respondents were either male or female, except one individual who chose ‘Prefer not to say’ who we excluded from the statistical test. We analyzed religious affiliation using a non-parametric Kruskal-Wallis rank sum test. We did not statistically evaluate survey response and self-described racial identity as all but four respondents reported being ‘white’ and three of the remaining four chose ‘Prefer not to say’. For a similar reason, we did not perform a statistical evaluation of highest level of academic achievement as all but one participant had earned a Ph.D. and the remaining participant had earned a Master’s degree.

To determine if self-selection bias may have played a role in the resulting demographics of study participants, we utilized available data on nationwide demographics of university professors and estimated, based on those studies, the approximate distribution of participants that we could have expected based on our total sample size (n=41). We took data on political party affiliation and ideology were taken from Gross and Simmons,^2^ their tables 3 (ideology, ‘total’ category) and 6 (political party). Data on religious affiliation and gender were taken from Ecklund and Scheitle,^3^ their tables 2 (gender) and 4 (religious identification). We chose these two sources as they are well-accepted and the categorical choices for each demographic characteristic were nearly identical to ours. It is worth noting that in using these two sources, we used average demographics of university professors across all fields. However, both studies partition some of their data (though not all) by subfield. In both studies, the subfield that most closely aligns with the population of experts here (physical/biological sciences in ^2^; and natural sciences/biology in ^3^) show demographic characteristics that are largely representative of university professors as a whole (i.e. within a few percentage points) for ideology, political party, and gender. Data on religious identification was not partitioned to a subfield level in ^3^. We also attempted to find data on demographic characteristics of US anatomy professors specifically for our reference data (e.g. ^4^), however data did not exist for all demographic variables, and for those for which it did exist, did not match our variables categorical scheme. We multiplied the proportions for each demographic category by our sample size and tested the resulting expected count data versus our actual data (Table 1) using Fisher’s exact tests for count data. We did not include religious level in these tests as we did not find adequately similar nationwide data. In no case were our demographic data significantly different that those that would have been expected based on nationwide trends of American college professors.

We performed all statistical analyses in R version 4.2.2^5^ and utilized the package ‘likert’.^6^

**Legends for Supporting Tables S1-S5.**

**Supporting Tables S1 (separate file).** Complete list of legislation investigated.

**Supporting Tables S2 (separate file).** Complete compilation of anatomical or embryological legislative facts.

**Supporting Tables S3 (separate file).** Initial statements grouped by category and type.

**Supporting Tables S4 (separate file).** Final statements and statistics.

**Supporting Tables S5 (separate file).** Full deidentified responses (Redcap labelled format)

**SI References**

1. Daniels CR, Ferguson J, Howard G, Roberti A. Informed or misinformed consent? Abortion policy in the United States. *J Health Polit Policy Law*. 2016;41(2):181-209.

2. Gross N, Simmons S. The social and political views of American professors. *CiteSeerX*. 2007;10.1.1.147.6141.

3. Ecklund EH, Scheitle CP. Religion among academic scientists: distinctions, disciplines, and demographics. *Soc Probl*. 2007;54(2):289-307.

4. Sumner DR, Hildebrandt S, Nesbitt A, Carroll MA, Smocovitis VB, Laitman JT, Beresheim AC, Ramnanan CJ, Blakey ML. Racism, structural racism, and the American Association for Anatomy: initial report from a task force. *Anat Rec*. 2022;305(4):772-87.

5. R Core Team. R: *A Language and Environment for Statistical Computing*. Vienna: R Foundation for Statistical Computing; 2022. Available from: http://www.r-project.org.

6. Bryer J, Speerschneider K. *likert: Analysis and Visualization Likert Items*. 2016. Available from: https://cran.r-project.org/package=likert.
